# Supplementary material for: Subjective nature of path information in quantum mechanics
Source: Nat Commun. 2026 Feb 6;17:2433. doi: 10.1038/s41467-026-69034-7 (PMC12988235; doi:10.1038/s41467-026-69034-7)
Supplement: Supplementary file 1 — Supplementary Information [file 41467_2026_69034_MOESM1_ESM.pdf]

Supplementary Information for "Subjective nature of path information in quantum mechanics"

Xinhe Jiang[1,2†], Armin Hochrainer[1,2†], Jaroslav Kysela[1,2], Manuel Erhard[1,2], Xuemei Gu[1,3], Ya Yu[1,4], Anton Zeilinger[1,2*]

[1]Institute for Quantum Optics and Quantum Information, Austrian Academy of Sciences, Boltzmanngasse 3, Vienna, 1090, Austria.
[2]Vienna Center for Quantum Science and Technology, Faculty of Physics, University of Vienna, Boltzmanngasse 5, Vienna, 1090, Austria.
[3]Max Plank Institute for the Science of Light, Staudtstraße 2, Erlangen, 91058, Germany.
[4]Shanghai Jiao Tong University, Dongchuan Road 800, Shanghai, 200240, China.

*Corresponding author(s). E-mail(s): anton.zeilinger@univie.ac.at;
†These authors contributed equally to this work.

Contents

Supplementary Notes 1-2

Supplementary Figures 1-12

Supplementary Table 1

Supplementary Note 1: Interference of three processes

In this section, we show that our setup is a nonlinear interferometer with three processes, and the interpretation of path information is indeed subjective. The nonlinear interferometer incorporates a SU(1,1) transformation and is fundamentally different from the traditional SU(2) Mach-Zehnder and Hong-Ou-Mandel-type interferometer [1]. It describes an *active* optical dynamics, which means that the ports of the interferometer can be left in vacuum. Therefore, one can treat our setup as three alternative processes that occur within each crystal as a result of second-harmonic generation. In Supplementary Figure 1, we plot the three processes. The first process I is from the pump laser to crystal NL1 and to the detector. The second process II is from the pump laser to the crystal NL2 and to the detector. The third process III is from the pump laser to the crystal NL3 and to the detector. Each crystal contributes to an alternative, that is, NL1 to $A = ae^{i\phi_A}|s\rangle|i\rangle$, NL2 to $B = b|s\rangle|i\rangle$, and NL3 to $C = ce^{i\phi_C}|s\rangle|i\rangle$. When treating the system as a whole, one accounts for all these terms and interprets the probability as $P_{\text{three}} = |A + B + C|^2$.

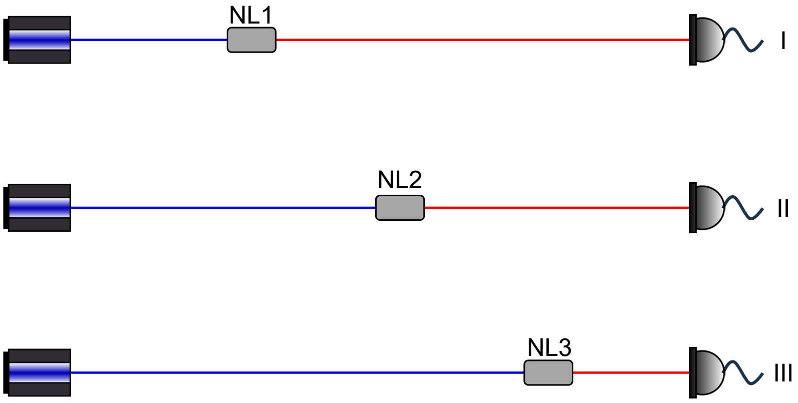

Supplementary Figure 1: Interference of three processes. The first process I is from the pump laser to NL1 and to the detector. The second process II is from pump laser to NL2 and to the detector. The third process III is from the pump laser to NL3 and to the detector. Each crystal contributes to one interference term, i.e., NL1 to $A = ae^{i\phi_A}|s\rangle|i\rangle$, NL2 to $B = b|s\rangle|i\rangle$, and NL3 to $C = ce^{i\phi_C}|s\rangle|i\rangle$.

In the case of two-crystals (NL1 contributes to $A = ae^{i\phi_A}|s\rangle|i\rangle$, NL2 contributes to $B = b|s\rangle|i\rangle$), one has $P_{\text{two}} = |A + B|^2$. Assuming $A = 0$, we have $P_{\text{two}} = |0 + B|^2 = CC$. An experimenter is asked to determine the origin of the detected photons. The experimenter can perform the experiment. They first remove NL1 or filter the SPDC photons from NL1 and find that the detectors have counts CC. Then, they move NL1 in or remove the filter and find that the counts are still the same. The experimenter also operates NL2 in the same way. They will find that the counts are zero when NL2 is removed and that the counts remain CC when NL2 is present. Thus, the experimenter concludes that the photons originate from NL2. This is in accordance with the mathematical meaning. Therefore, one can have a high probability of knowing where the photons come from if one source emits no photons in the two-crystal case. The mathematical meaning and the final physical interpretation, based on the observation of the experimenter, are consistent.

In the three-crystal case, different experimenters can arrive at different conclusions for the same underlying setup. Conceiving the Gedankenexperiment (see Supplementary Figure 2): Two experimenters (E1 and E2) receive a setup composed of two black boxes (BB1, BB2), and a phase shifter is inserted between these two boxes. For E1, NL1&NL2 is placed into BB1 and NL3 is in BB2. No photons are emitted from NL1&NL2. For E2, NL1 is placed into BB1 and NL2&NL3 is in BB2. No photons are emitted from NL2&NL3. Similarly, the experimenters are asked to determine the origin of the photons. Based on their experience in the two-crystal case, the experimenters can perform a similar operation by treating each black box as a source. First, they remove BB1 and then reinsert it; second, they remove BB2 and then reinsert it. After this, experimenter E1 comes to the conclusion that under these observations, the probability amplitude for photon-pair generation in crystals NL1&NL2 does not contribute to the detected events. Consequently, conditioned on detection, the

photon pairs can be operationally attributed to crystal BB2=NL3. Whereas experimenter E2 concludes that the photons can be attributed to crystal BB1=NL1 based on their observation. One sees that fundamentally both experimenters have the same experimental setup. The different conclusions they obtained are simply because they conceptualize the experiment differently, or the experimental setup manifests itself differently. These two perspectives just correspond to the two different mathematical possibilities, i.e., $P^{(1)}_{\text{three}} = |0 + C|^2$ and $P^{(2)}_{\text{three}} = |A + 0|^2$, if $\phi_A = \phi_C = \pi$ and $|A|^2 = |B|^2 = |C|^2$. Here, one can see that interpretations of path information are highly subjective. It depends on how one conceptualizes the experiment's configuration. Furthermore, if a third experimenter finds that one black box has two crystals and opens the black boxes, they will interpret that all three crystals contribute to the counts if they are quantum physicists and believe in quantum rules. Or, in an ingenious way, they can say that the photons come from the system as a whole. In the case of two crystals, there is no inconsistency in the interpretation of the path information. The inconsistency and subjectivity in the interpretation of path information emerge only in the three-crystal case.

Supplementary Note 2: Apply duality relation in our setup

Here we show that the duality relation can be applied directly to our setup. For interferometry with two crystals, the photon pair emission rate is described by an interference law analogous to the two-path interferometer, i.e., $R \propto |a + e^{i\phi}b|^2 \propto a^2 + b^2 + 2ab\cos\phi$, where a and $e^{i\phi}b$ are the corresponding probability amplitudes contributed by these two crystals, and ϕ is the relative phase between these amplitudes. To apply the duality relation to our setup, we define the interference visibility V similar to the traditional two-path interferometer

$$V = \frac{R_{\max} - R_{\min}}{R_{\max} + R_{\min}} = \frac{2ab}{a^2 + b^2} \tag{1}$$

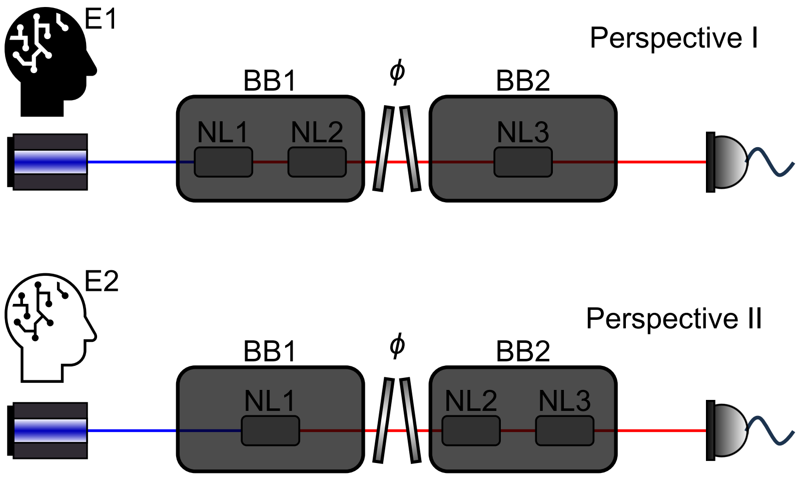

Supplementary Figure 2: Two perspectives to interpret the experiment. Perspective I: NL1&NL2 is placed into black box 1 (BB1) and NL3 is placed into BB2, and no photon is emitted from NL1&NL2. Perspective II: NL1 is placed into BB1 and NL2&NL3 is placed into BB2, and no photon is emitted from NL2&NL3. Each experimenter performs the following experiment: They first remove BB1 and then reinsert it; secondly, they remove BB2 and then reinsert it. E1: Experimenter 1. E2: Experimenter 2.

The distinguishability D in this case corresponds to the distinguishability of "which-source" produced a photon pair, and can be quantified in a similar way as in the traditional two-path interferometer [2]

$$D = \frac{a^2 - b^2}{a^2 + b^2} \tag{2}$$

With this, the duality relation can be directly used in our setup. The "which-source" information is simply that the photon pair is produced at which source and travels all the way to the detector. When full path information is available, it means that one knows where the photon pair originated.

Supplementary Table 1: Experimentally obtained duality relations.

	V	$D = p3^2/p1^2$	V^2	D^2	$D^2 + V^2$
S1&S2	0.0912	0.9514	0.008317	0.905162	0.913479
S1′&S2′	0.0830	0.9641	0.006889	0.929489	0.936378

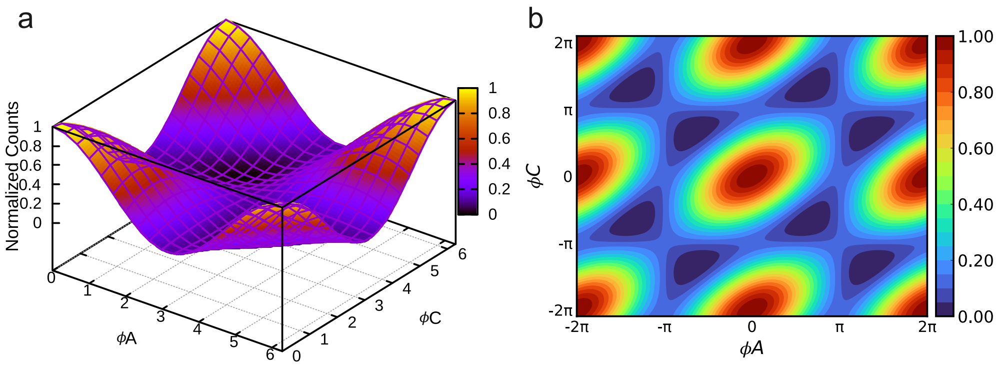

Supplementary Figure 3: a, Count rates on varying phase ϕ_A and ϕ_C obtained with equation (2) in the main text. The range is from 0 to 2π. b, Theoretical contour plot in the range from -2π to 2π. Equal emission probabilities a, b, and c are assumed. At the point $\phi_A = \pi$ or $\phi_C = \pi$, one can see that interference visibility is zero.

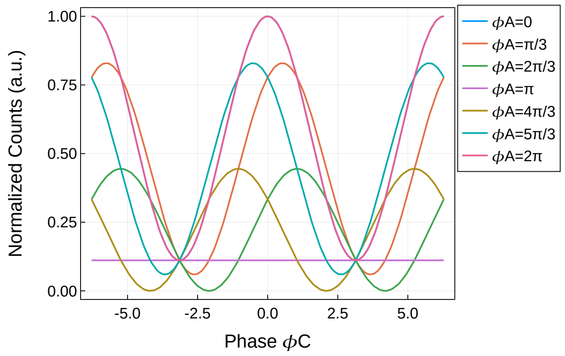

Supplementary Figure 4: Count rates versus phase ϕ_C for several fixed phases $\phi_A = 0, \pi/3, 2\pi/3, pi, 4\pi/3, 5\pi/3, 2\pi$. Note that the highest visibility curve has a lower count rate and the middle visibility curve has a higher count rate.

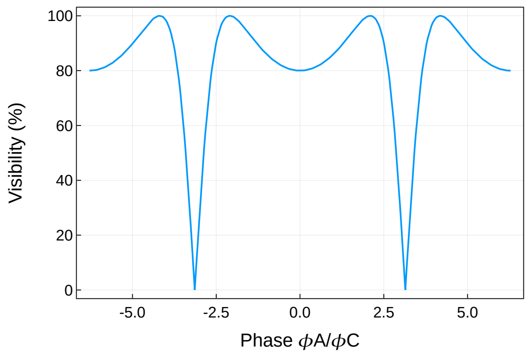

Supplementary Figure 5: Interference visibility between the three crystals. The range is from -2π to 2π. Three particular points are interesting for us to compare, i.e., phase 0, phase $2\pi/3$ of highest visibility 1, and phase π of zero visibility.

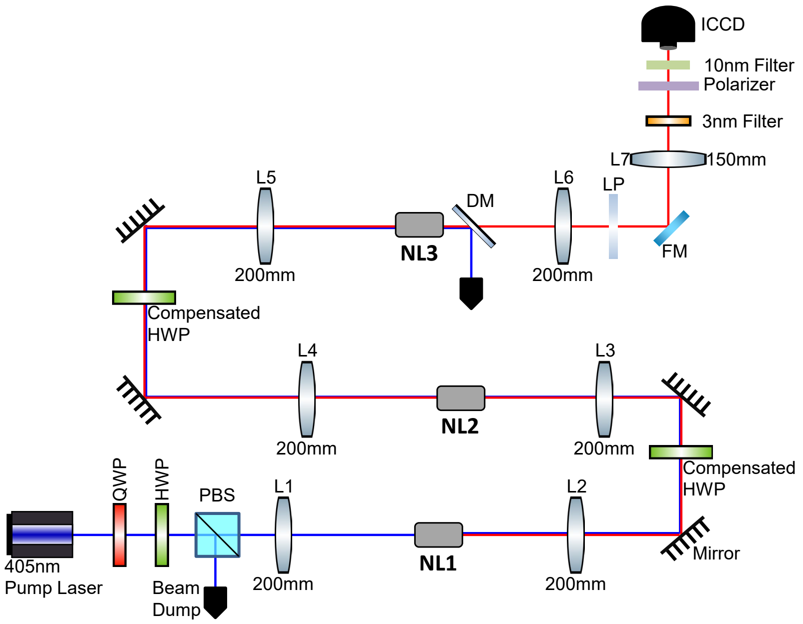

Supplementary Figure 6: Setup for the imaging of the SPDC photons from the three crystals. The setup before the flop mirror (FM) is almost the same as in the main text; only the phase plate is not included. After FM, we used an imaging system to image the SPDC photons on the ICCD camera.

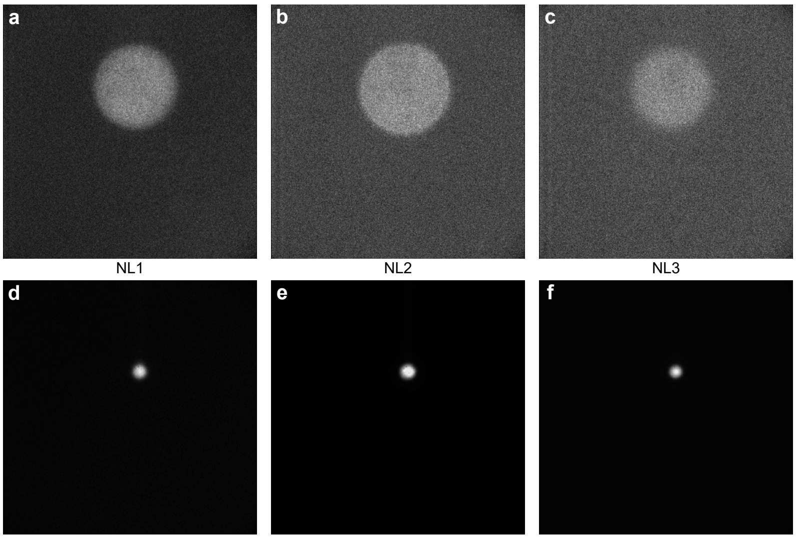

Supplementary Figure 7: Image of the SPDC photons from the three crystals with an ICCD camera. a-c are for the Fourier plane. d-f are for the crystal plane.

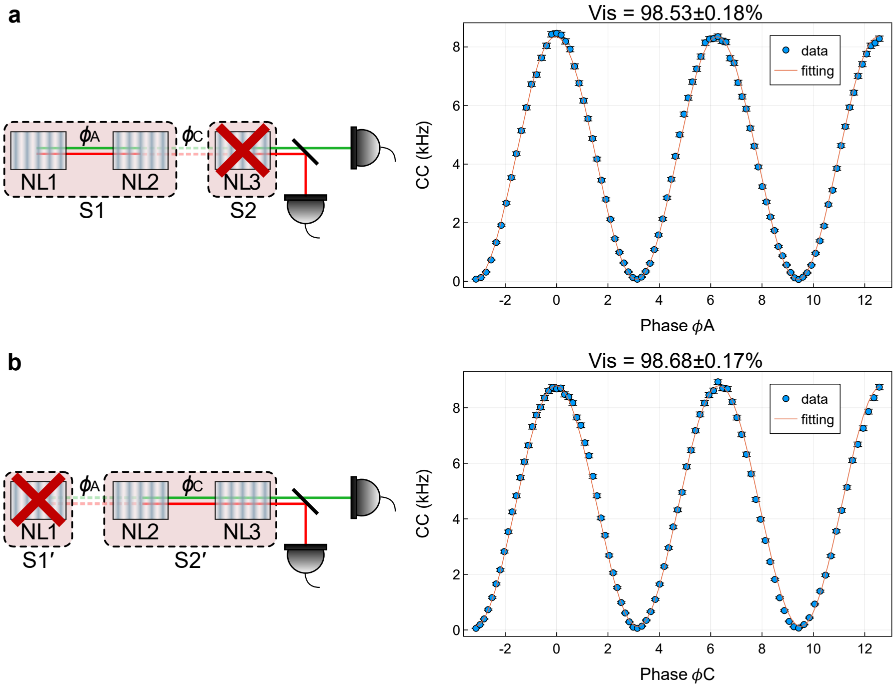

Supplementary Figure 8: Interference between each pair of crystals. a, Left: Grouping of NL1 and NL2. Right: Coincidence counts (CC) versus relative phase ϕ_A when NL3 is blocked. The interference visibility obtained between NL1 and NL2 is 98.53±0.18%. b, Left: Grouping of NL2 and NL3. Right: CC versus the relative phase ϕ_C when NL1 is blocked. The interference visibility obtained between NL2 and NL3 is 98.68±0.17%. The data are fitted with a sinusoidal curve. Errors are determined by assuming Poissonian counting statistics.

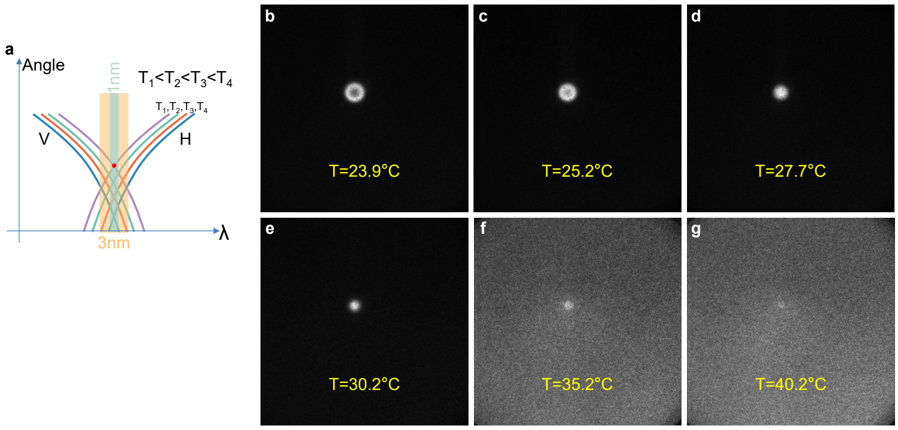

Supplementary Figure 9: a, Angle distribution of the SPDC photons versus the wavelength at different crystal temperatures. This is an ideal case. Normally, the central wavelength of the band-pass filter is not the same as the degenerate wavelength of the SPDC photons. In addition, the photons at 0° angle are not within the bandwidth of the filter. Therefore, we usually collect photons located at the rings filtered by the band-pass filter. b-g, Variation of the SPDC beam spot when changing the crystal temperature. In the experiment, the crystal temperature is optimised around 30°C.

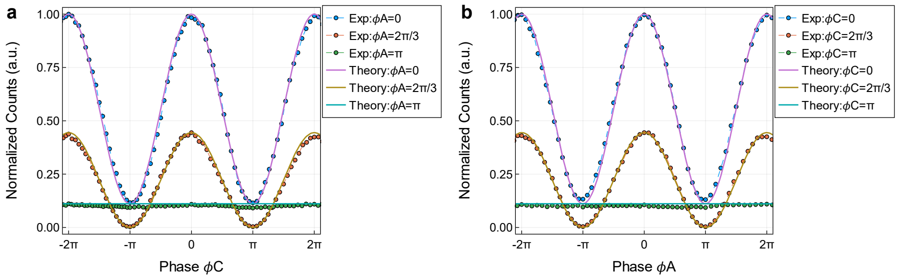

Supplementary Figure 10: Count rate versus the phase ϕ_C (a) and phase ϕ_A (b). For comparison, the experimental counts are normalised with respect to the corresponding theoretical maximum.

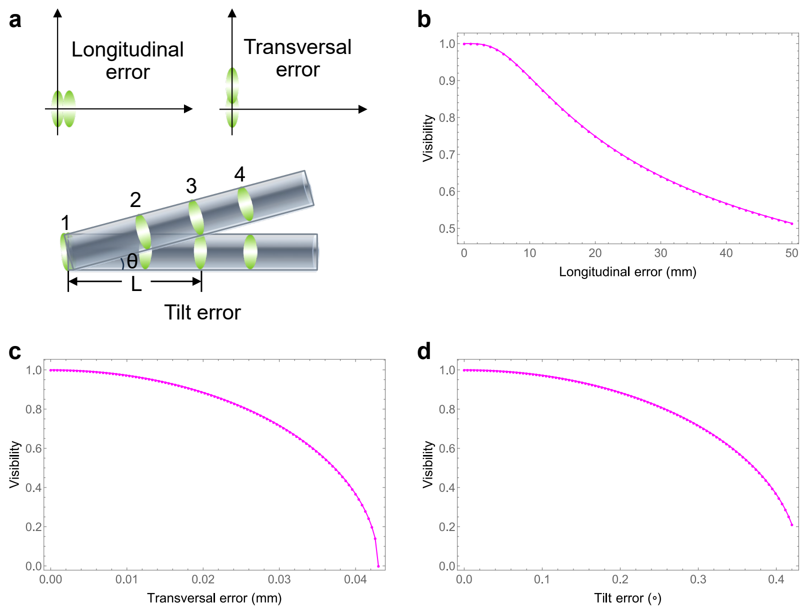

Supplementary Figure 11: Visibility degradation caused by three different kinds of alignment errors. a, Schematic of the three errors. b, Visibility versus longitudinal error. c, Visibility versus transverse error. d, Visibility versus the tilt error.

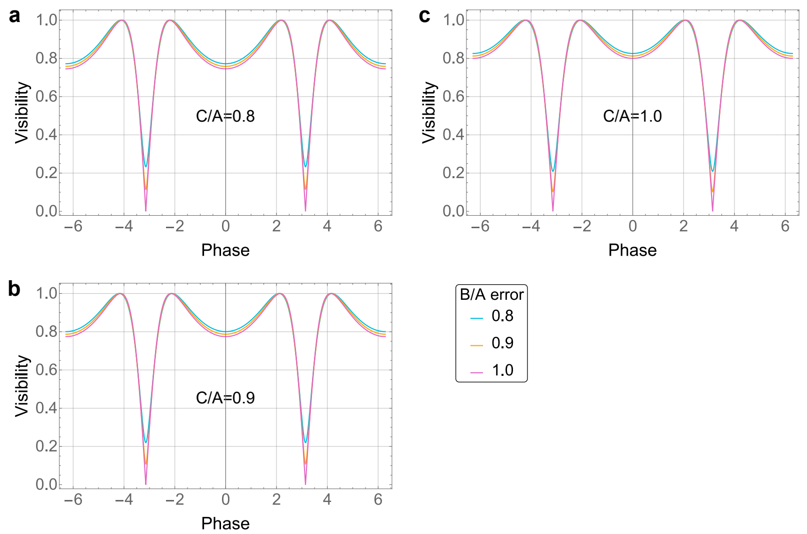

Supplementary Figure 12: Visibility deviation due to the photon yield imbalance of the three crystals. a, b and c are for the different imbalance ratios C/A between NL3 and NL1. The three lines in each figure correspond to the different imbalance ratios $B/A = 0.8, 0.9, 1.0$ between NL2 and NL1, as shown in the legend. Here, the abscissa is the phase ϕ_A. The results are the same for phase ϕ_C.

References

[1] Yurke, B., McCall, S. L. & Klauder, J. R. Su(2) and su(1,1) interferometers. *Phys. Rev. A* **33**, 4033–4054 (1986).

[2] Greenberger, D. M. & Yasin, A. Simultaneous wave and particle knowledge in a neutron interferometer. *Phys. Lett. A* **128**, 391–394 (1988).
